# Tailoring the Chicago Parent Program for Foster and Kinship Caregivers: a Mixed Methods Approach

**DOI:** 10.1007/s11121-025-01797-9

**Published:** 2025-03-01

**Authors:** Sarah J. Beal, Nathan Lutz, Meera Patel, Julie Dougherty, Ro Gigger, Lisa M. Vaughn, Mary V. Greiner, Amie F. Bettencourt, Susan M. Breitenstein, Debbie Gross, Robert T. Ammerman

**Affiliations:** 1 Cincinnati Children’S Hospital Medical Center and the Department of Pediatrics, University of Cincinnati College of Medicine, Cincinnati, OH USA; 2 Cincinnati Children’s Hospital Medical Center, Cincinnati, OH USA; 3 Department of Psychiatry and Behavioral Sciences, Johns Hopkins University, Baltimore, MD USA; 4 College of Nursing, The Ohio State University, Columbus, OH USA; 5 School of Nursing, Johns Hopkins University, Baltimore, MD USA

**Keywords:** Prevention, Child welfare, Foster care, Parenting program

## Abstract

There is a lack of evidence-based programs to support the prevention of child behavior problems designed specifically for foster and kinship caregivers from historically minoritized groups. Drawing on existing best-practice recommendations for tailoring interventions to new cultural and social contexts, this study evaluates initial evidence of the acceptability and feasibility of adapting the Chicago Parent Program (CPP) for foster and kinship care. An expert panel of foster and kinship caregivers, clinicians, and other professionals with experience in CPP and child welfare arenas was convened to review and adapt CPP materials in collaboration with researchers. The adapted program was then delivered to 12 foster and kinship caregivers, who completed surveys and qualitative interviews to provide feedback about the revised program. Caregiver satisfaction with the program was generally high. In interviews, caregivers emphasized that group sessions resonated with them (theme 1), that they appreciated the opportunity to connect with the program via mandated foster care clinic visits (theme 2), and that adapted components of the program delivery (theme 3), and materials (theme 4) were relevant. Caregivers also noted barriers with behavior management and logistical challenges that made it more difficult to attend regularly and complete practice assignments, which was reflected in reduced participation rates during the pilot. Overall, these findings suggest that careful and empirically driven adaptation to effective parent-focused prevention programs can be achieved for foster and kinship caregivers, leading to high potential impact for a population that has generally been under-resourced and experiences high need and mental health burden.

**Supplementary Information:**

The online version contains supplementary material available at 10.1007/s11121-025-01797-9.

## Tailoring the Chicago Parent Program for Foster and Kinship Caregivers

There is robust evidence to support parenting programs to prevent child behavior problems and bolster mental and behavioral health in young children (Bater & Jordan, 2017; Healey & Fisher, 2011). Programs include didactic-based curricula in individual or group settings (Blair et al., 2019; Chamberlain, 2003), home visiting (Alves et al., 2024), and dyadic parent–child interventions (Blair et al., 2019; Mersky et al., 2016). These programs provide parents with tools and strategies to support parent–child attachment (Wright & Edginton, 2016), communicate with children (Johnson et al., 2005), and establish consistent rewards and consequences that are known to modify behavior (Forehand et al., 2013). Each of these aspects of parenting programs has been robustly studied (Eyberg et al., 2008, 1999) and demonstrates strong evidence of effectiveness in changing children’s behavior individually and in combination (Jeong et al., 2021).

Parenting programs have demonstrated benefits in increasing parent self-efficacy and confidence in parenting, and in reducing stress (Bloomfield & Kendall, 2012; Fang et al., 2021; Wittkowski et al., 2016). Both parenting stress and parent self-efficacy are associated with better parent–child relationships (Albanese et al., 2019; Çekiç & Karageyik, 2021). In other words, preventative parenting programs impact child behavioral health directly through strengthened parent–child interactions and indirectly through support for parents.

Prevention programs designed for parents reflect the cultural and social contexts of the parent–child dyads they were developed with, which have implications for how effective they are in supporting diverse families. An emphasis on dyadic interactions between a single parent and child, for example, may be acceptable and feasible for a two-parent household with few children and unacceptable for households and cultural groups where the incorporation of multiple children in the process of socialization is more common (Mersky et al., 2016). Likewise, monitoring for adherence to parenting behaviors may be perceived as intrusive in communities that have experienced historical trauma related to surveillance (Mejia et al., 2017; Smith et al., 2022; Toure et al., 2010). For these reasons, multiple efficacious parent-focused prevention programs are available for use across settings (Albritton et al., 2024; Christensen et al., 2024).

Almost all parenting programs assume that the parent participating is a mother or father to the children benefitting from the program. There is good reason for this: the majority of children in the USA, for example, are raised by at least one biological or adoptive parent (Radel et al., 2016). However, for the 3.1% of children residing in non-parental care, the social and contextual factors that influence the caregiver-child relationship are often more complex (Beal & Greiner, 2016) and do not reflect the families for whom preventive parenting programs have been developed. For example, in a recent review of parenting programs, only 29% were found to include any evidence of effectiveness with foster caregivers (Dalgaard et al., 2022). Likewise, the California Evidence-based Clearinghouse (CEBC; 2024) lists 11 programs focused on child behavior and wellbeing as effective or promising for parents of children ages 2–8. Of those, 10 programs were supported for delivery to foster and kinship caregivers. Importantly, only four programs available to foster and kinship caregivers were rated by CEBC as having high child welfare relevance, and only one was supported by research evidence (Supplemental Table 1).

### The Need to Tailor Interventions to Foster and Kinship Contexts

Over 430,000 children are in child welfare protective custody (i.e., foster care) in the USA (U.S. Department of Health and Human Services, 2020). Children in foster care are disproportionately from families with low incomes (Barth et al., 2006; McGuinness & Schneider, 2007). Children from racial and ethnic minority backgrounds are over-represented (Maloney et al., 2017), and more than half enter foster care from urban settings (Wulczyn & Hislop, 2002).The primary goal of foster care is to ensure child safety and support family preservation (Chambers et al., 2018); safety is addressed by placing children with temporary caregivers (i.e., kinship or licensed foster caregivers) with caseworker oversight (Park & Helton, 2010).

There are several reasons to consider foster and kinship caregiver-child relationships as unique social contexts in delivering preventive parenting programs. First, children in foster and kinship care are exposed to maltreatment or other risks (e.g., domestic violence) that compromise child safety (Jee et al., 2010; Turney & Wildeman, 2017). These exposures are linked to increased internalizing and externalizing behavior problems in children (Beal et al., 2018; Schroeder et al., 2020)—representing unique mechanisms of risk for child behavior. Second, children in foster and kinship care are separated from their parents, which impacts attachment (Barth et al., 2006; Hunt et al., 2017; Jee et al., 2010; Schroeder et al., 2020; Turney & Wildeman, 2017; Yates et al., 2003; Zima et al., 2000) and other aspects of the parent–child relationship that foster and kinship caregivers are expected to manage (e.g., supporting visitation between children and their parents to facilitate future reunification). Third, the role of the foster and kinship caregiver as a substitute “parent” often results in inconsistencies across expectations and communication styles, even when the caregiver is a relative. The caregiver’s limited knowledge of historical interactions between parents and children contributes to inconsistent and unclear caregiver-child communication. These contextual factors are important to address when parenting programs are delivered to foster and kinship families. Unfortunately, the work to adapt evidence-based parenting programs to foster and kinship care is limited.

### Adapting Evidence-Based Programs for Delivery in New Contexts

Card et al. (2011) outline seven steps to program adaptation for a new context: 1—selecting a suitable program; 2—gathering original program materials; 3—developing a program model; 4—identifying the program’s core components and best practice characteristics; 5—identifying mismatches between the original program model or materials and the new context; 6—adapting the original program model; and 7—adapting the original program materials. Below we describe the process of tailoring the Chicago Parent Program (CPP) for foster and kinship care, with evidence of the effectiveness of the adaptation process.

### Steps 1–4: The Original Program

#### Steps 1 and 2: Selection of a Suitable Program and Collection of Original Materials

The CPP was selected because it is evidence-based and was developed in collaboration with an advisory board of African American and Latino families from under-resourced communities to ensure its cultural and contextual relevance (Bettencourt et al., 2024; Breitenstein et al., 2012; Gross et al., 2009). The CPP addresses challenges faced by caregivers who are working with children in foster and kinship care (e.g., attachment, communicating expectations, rewards and consequences, addressing caregiver stress and confidence; Dalgaard et al., 2022). Foster and kinship caregivers who completed the CPP in its original format (*n* = 5) found it to be acceptable and identified areas for adaptation, including the need to include more examples that reflect foster and kinship caregivers experiences with children’s behaviors, including group leaders with knowledge of foster and kinship care, enhancing accessibility, and discussing the application of program concepts to experiences (e.g., placement changes) common in foster care (Freehling et al., 2025). The developers of the CPP were amenable to tailoring it for foster and kinship care with an executed memorandum of understanding. All available CPP materials were provided to support the adaptation of the program to foster and kinship care and further facilitate the application of the Card et al. (2011) approach to adapting evidence-based programs for new contexts. This included the group leader manual, fidelity materials, and course materials (e.g., handouts, videos).

#### Steps 3 and 4: Develop the Program Model and Identify Program Core Components

The CPP has an established logic model (Breitenstein et al., 2020; Gross, n.d.) that outlines the program components and goals, strategies and principles, risk and protective factors influenced by the strategies and principles, and outcomes. Core components include having two group leaders who are trained to ensure implementation fidelity, ground rules to establish a safe learning environment, the use of group discussions and video vignettes to stimulate learning, the use of role plays and group activities to develop new skills, and weekly handouts and practice assignments to further develop skills. During group sessions, parents establish goals, receive group support, and develop new skills to address the parent–child relationship, behavior management, and effective coping and problem-solving strategies. Group leaders are trained to support the expertise of parents who are accomplishing the parents’ goals while facilitating skill development through video modeling and group discussion. Those strategies increase parents’ confidence in managing children’s behaviors and consistency with implementing evidence-based behavior management strategies, reduce parents’ stress, and promote collaboration between parents and other adults to support better child behavior outcomes.

### Current Study

This study builds upon prior work that established the CPP as an evidence-based prevention program (Breitenstein et al., 2020) acceptable to foster and kinship caregivers (Freehling et al., 2025). The purpose of this study is to describe the process by which the CPP was tailored to the foster and kinship care context and metrics to assess the success of that adaptation process, with a focus on acceptability and satisfaction. It was expected that by following a rigorous model for adaptation, there would be robust evidence of the program’s acceptability from foster and kinship caregivers without the need to adapt the program further.

## Methods

### Context

All US states require health screenings when children enter foster care (Thompson, 2021). In Ohio, children are required to have health examinations when they enter care and with each placement change. The CHECK (Comprehensive Health Evaluations for Cincinnati Kids) Foster Care Center at Cincinnati Children’s Hospital conducts mandated healthcare examinations for children in southwest Ohio. Clinical research staff are embedded into the clinic to recruit for research studies and have access to a shared health and child welfare administrative database to use for recruiting and retaining families in research studies. The CHECK Center includes a diverse team trained in clinical and experimental psychology, medicine, nursing, social work, and education. Several staff members have experience as foster caregivers.

### Procedures

#### Program Adaptation

A foster and kinship caregiver advisory board of two kinship and four licensed foster caregivers, as well as one young adult with lived experience in foster care, was assembled for this study. Advisory board members were diverse in age (29–66 years), race and ethnicity (57% Black or African American, 14% Hispanic), and education (high school only to postdoctoral training). Research and advisory board members reviewed feedback from caregivers who had previously completed CPP (Freehling et al., 2025) as well as all CPP materials to identify areas for tailoring. Over a series of five meetings, the group collectively read through the CPP manual, handouts, and related materials and members were asked to flag language, concepts, or other components of the intervention that they or other caregivers might desire changes to. When a change to materials was identified, the group discussed adaptation strategies until agreement was met. Between meetings, the investigative team would make proposed changes to materials, which were reviewed by the advisory board at the next meeting. Advisory board members were compensated $250 each for their efforts. This study received institutional approval from the local Institutional Review Board; data are available upon reasonable request to the corresponding author.

##### Step 5: Identifying Mismatches Between the CPP and Foster and Kinship Care Context

Table 1 summarizes the misalignment between the CPP model and materials and foster and kinship care contexts, including by whom the misalignment was identified. Most issues were first identified by the investigators and advisory board. This included the need to add information that would provide group leaders who facilitate the CPP with sufficient background to deliver materials effectively to foster and kinship caregivers, as well as to the terminology used, how some core concepts were presented, and the approaches that caregivers might take in establishing relationships and communicating expectations with the children in their homes.

**Table 1:** Summary of misalignment between CPP and the foster and kinship care context

Misalignment	How identified	Tailored resolution
Logic model		
Group leader training does not address foster and kinship care contexts	Investigators, advisory board	The introduction and “For Group Leaders Only” sections were added to address specific issues/knowledge gaps in foster and kinship care. The Advisory board also met with study staff to review preferred terminology for these services. They also helped add some trauma-informed language to the group leaders’ boxes
Description of weekly sessions, including time commitment, predictable breaks, caregiver interactions, debriefing, and flexible scheduling	Caregivers, advisory board	Details about time commitment and strategies for making sessions effective added, more time for caregiver interactions and debrief in sessions added, scheduled breaks added; concurrent sessions to allow caregivers to join more than one class time, note from lead investigator about child distractions and approval/support in stepping away from the session to manage child behavior/other things that may come up
Role plays do not include foster/kinship terms and scenarios (e.g., interactions with caregivers and family of origin)	Investigators, advisory board	Select role-play scripts and scenarios adapted to include diverse terms and scenarios for foster and kinship care
Practice assignments do not include foster/kinship terms and scenarios (e.g., interactions with caregivers and family of origin)	Investigators, advisory board	Select practice assignments and scenarios adapted to include diverse terms, scenarios for foster and kinship care
Handouts do not include foster/kinship terms and scenarios (e.g., interactions with caregivers and family of origin)	Investigators, advisory board	Select handouts adapted to include diverse terms and scenarios for foster and kinship care
Caregivers do not have long-term roles in children’s lives, which is counter to the CPP philosophy that parents should set long-term goals for children	Investigators, advisory board	Short-term goals and long-term abstract ideas identified by caregivers were reframed to support that caregivers make a long-term difference even with short-term involvement
Caregivers do not know children well, which is counter to the CPP philosophy that parents are experts	Advisory board	Re-frame that caregivers are experts with skill selection, becoming experts for the new child in their home
Risk factors unique to foster and kinship care not identified	Investigators, advisory board	Introduction, “For Group Leaders Only” sections added to address specific risk factors for foster and kinship care
Protective factors and motivators unique to foster and kinship care not identified	Investigators, caregivers, advisory board	Introduction, “For Group Leaders Only” sections added to address specific protective factors for foster and kinship care; allocation of training hours for licensure added
Program outcomes unique to foster and kinship care not identified	Investigators, advisory board	Placement stability identified as foster and kinship care, teach caregivers stress management strategies, help build connections and relationships with children in the first months of placement
Materials		
Routines and rituals (CPP session 2) are not in place to support parent–child interactions (CPP session 1)	Investigators, advisory board	Session order changed (sessions 1 and 2 swapped) to start with establishing routines
Terminology (Parent, your child) did not reflect relationships between caregivers and children	Investigators, advisory board	Terms modified to “Caregiver” and “the children in your home”
Routines and traditions may differ for caregivers and the children in their home, may need to reflect children’s histories	Investigators, advisory board	Activities added to solicit routines from family of origin, children, support incorporating those routines into the caregiver’s household
Time-out and physical discipline policies are different in foster and kinship care	Investigators, advisory board	Background information to contextualize discussions about time-outs, physical discipline were added. Materials were adapted to support discussion around time-out and to reinforce that physical discipline is not permitted
Concern that guidance regarding ignoring behaviors may be ineffective if the child is not attention-seeking	Investigators, advisory board	Clarification that ignoring is paired with a statement explaining what behavior the caregiver wants the child to engage in
Concern that behaviors were not reflective of the full spectrum of behaviors observed for children in foster and kinship care	Investigators, advisory board	Additional child behavior scenarios added, worksheets and activities modified to include more extreme child behaviors common in foster and kinship care

##### Step 6: Adapting the CPP Model

While many of the core components of the CPP remained the same, several aspects of the program’s logic model (Breitenstein et al., 2020; Gross, n.d.) required tailoring to the foster and kinship care context. This was primarily accomplished by developing a Guide for the Delivery of the CPP to Foster and Kinship Care, which was created in collaboration with and approved by the CPP developers. The guide includes detailed information about the foster and kinship care context to ensure that group leaders who facilitate weekly sessions are knowledgeable about foster and kinship care, with additional information added or highlighted using “For Group Leaders Only” boxes in the session-by-session guide. The advisory board also recommended that some aspects of the program logic model (e.g., parents as experts, parents’ roles in setting goals for their children, types of behaviors being managed) be modified to better represent the foster and kinship care experience, where caregivers often do not have long-term plans for the children in their homes and do not know children well enough to perceive themselves as experts in managing the specific child’s behaviors. The unique risk and protective factors that foster and kinship caregivers and the children in their homes face were also identified as needing adaptation, which was accomplished by contextualizing some of the discussion questions and group-based activities delivered as part of the CPP. Finally, the logistics of delivering the CPP over 12 weeks was identified as a concern with solutions that may be similar to the delivery of CPP in other settings. The delivery of the CPP-FC online alleviated challenges with caregivers needing transportation to a specific place at a certain time. Providing resources (e.g., pizza delivery, play items to keep children occupied at home during groups), flexible scheduling of sessions (e.g., caregivers could flex between a group meeting on a weekday evening and a group meeting on a Saturday morning), and multiple communications and reminders ahead of weekly sessions (e.g., text messages and emails alongside calendar invites sent the week prior, day prior, and day of sessions) were included in the guidelines for the delivery of the CPP-FC.

##### Step 7: Adapting the CPP Materials

Six categories of modifications to the CPP materials were identified, primarily by the investigators and advisory board members, that resulted in tailoring of existing program materials (e.g., order of sessions, discussion questions used, handouts). The first two general program material changes were related to the order of sessions, which allowed caregivers to set up new routines with the children in their homes as a precursor to strategies to strengthen interactions between caregivers and children, and diversifying the terms used to refer to parents and children to reflect the foster and kinship care context. The third category of changes was to incorporate interactions with the families of origin into course practice assignments for children in out-of-home care. The remaining changes to program materials were to contextualize behavior management strategies to fit the experiences of foster and kinship caregivers, including incorporating common trauma-related behaviors into role-play scenarios, acknowledging trauma-informed components and misconceptions of time-out strategies, and supporting caregivers in communicating expectations to children new to their homes while also using ignoring strategies taught by the CPP. These adapted materials are fully detailed and included in the Guide for Delivery of the CPP-FC, a companion guide for group leaders trained in the CPP. The success of materials adaptation was reflected in both the survey data from study participants and their feedback during interviews.

#### Evaluation of Adaptation Effectiveness

The CPP adapted for Foster and kinship Care (CPP-FC) was delivered to caregivers recruited at the CHECK Center. Eligibility included being a kinship or licensed foster caregiver to a child ages 2–8 years who was presenting to the CHECK Center for a mandated new or change of placement exam, required within 5 business days of placement. Caregivers were told about the study by members of the study team.

Those who expressed interest consented to participate and completed baseline surveys, weekly practice assignments and satisfaction/acceptability surveys after each CPP-FC session, and a final survey to assess program acceptability and satisfaction. Between the primary sessions (T1–T11) and graduation booster session (T12), qualitative interviews were conducted with caregivers to provide feedback on the program and see where any additional changes needed to be implemented (Supplemental Table 2).

Baseline surveys took approximately 15 min to complete. Practice assignments took approximately 6 min to complete. Final surveys took approximately 20 min to complete. All surveys and practice assignments were collected via REDCap, which is an online data collection tool. Participants could complete REDCap surveys in clinic using our tablet and Wi-Fi or on their personal electronic devices if those were connected to Wi-Fi or were data-enabled. Qualitative interviews lasted 45 min, on average, and were conducted, recorded, and initially transcribed using Microsoft Teams. Interviews were completed by a member of the study team (MP) who has qualitative research training and was coached by a PhD-trained qualitative methodologist (LV). Caregivers could earn up to $155 for completing all study activities and up to 24 h toward ongoing training to maintain their certification as foster caregivers.

### Participants

Fifty-four caregivers were approached about participating in the CPP-FC and 23 enrolled. Primary stated reasons for the decline were time constraints (48%), lack of interest (13%), and anticipated placement changes (6%). Approximately half (52%) of caregivers completed at least one session of the CPP-FC, making them eligible for qualitative interviews and satisfaction surveys after each session and at the end of the program**.** Ten caregivers completed at least one weekly survey. Eight enrolled caregivers completed interviews; when enrolled caregivers had also included their partners or another adult in the home in CPP-FC sessions (i.e., household participation), the other adult was also invited to complete interviews (*n* = 4), resulting in 12 caregivers from eight households who completed interviews.

### Measures

#### Acceptability and Satisfaction

Session satisfaction questions from the CPP (six items) assessed aspects of the training materials, approach to program delivery, and group leaders using a Likert-response scale (1 = not helpful, 4 = very helpful). Caregivers were also asked specifically about the applicability of each session to them as foster or kinship caregivers, on a scale of 1 (not at all applicable) to 10 (very applicable). Program satisfaction questions from the CPP (four items) assessed overall satisfaction with the program (1 = very dissatisfied, 4 = very satisfied), as well as more general perceptions of aspects of the training materials using a Likert-response scale (1 = not helpful, 4 = very helpful). Finally, caregivers were asked “Would you recommend this program to a friend or relative?” with responses from 1 (would not recommend) to 3 (highly recommend).

### Analysis Plan

This study incorporated structured (i.e., from survey questions) and semi-structured (i.e., from interviews) data to evaluate the adaptation of the CPP-FC. Descriptive and summary statistics were calculated for satisfaction measures. Thematic analysis of qualitative data followed steps previously described by Braun and Clarke (Braun & Clarke, 2006): familiarization with the data, generation of initial codes, searching for themes, reviewing themes, and defining and naming themes. We used a hybrid inductive and deductive approach to code and analyze semi-structured interviews of program participants (DeJonckheere et al., 2024; Fereday & Muir-Cochrane, 2006; Proudfoot, 2023; Swain, 2018). Specifically, after we conducted inductive coding, we employed a deductive approach to mapping preliminary themes to steps 5–7 of the adaptation model described by Card et al. (2011) and then generated the final themes.

#### Inductive Coding

We began with an inductive analysis (bottom-up, data-driven) in which five team members read two interviews and shared preliminary codes they identified in the data. These preliminary codes were organized into an official codebook, which was adjusted to accommodate new codes as needed. The remaining interviews were coded by team member dyads. Code discrepancies were resolved through discussion. The full team met after groups of three interviews were coded to address any conceptual issues that emerged during the analysis.

#### Deductive Mapping

Following this inductive coding phase, a deductive approach (theoretical, top-down) was applied to map the inductive analysis onto the Card et al. (2011) framework. Four members of the coding team reviewed the codebook to identify components of the adaptation framework, including when participants expressed that the adaption was useful, when it was not useful, and when they wished for more or different experiences to support their learning.

#### Theme Generation

Theme generation followed an iterative process common in hybrid analyses, cycling between synthesizing the data according to the Card et al. (2011) framework and inductively analyzing transcripts. This process facilitated the synthesis of the data to inform the generation and naming of themes (Berkowitz, 1997; Proudfoot, 2023; Swain, 2018).

## Results

### Descriptive Statistics

Caregivers who participated in CPP-FC (*N* = 12) were women (75%) or men (25%), white and non-Hispanic (33%) or black and non-Hispanic (50%), foster caregivers (83%), and between 27 and 81 years of age (*M* = 50, *SD* = 17). Those caregivers who completed at least one survey (*n* = 10) did not differ demographically from those who participated in the CPP-FC, and caregivers who completed interviews (*n* = 8) were also demographically similar to caregivers who attended at least one session. Across 12 caregivers, 33% attended all sessions, 25% attended between 5 and 11 sessions, 33% attended between 2 and 4 sessions, and 8% attended only 1 session. Each week, an average of six caregivers submitted practice assignments (50%), with a range between 4 (33%) and 9 (75%) of practice assignments completed for each session.

### Evaluating Program Adaptation

#### Theme 1: Group Session Structure and Leadership Resonated with Caregivers

In qualitative interviews, caregivers reported that group interactions were their favorite parts of the sessions. Caregivers appreciated the space for exchanging ideas with peers.

You could talk freely... You know, if you have a certain question that you want to ask, you just say, may I have your permission to ask this and that? And I’m sure they would say yes… It stood out that people just talk freely. We didn’t hold anything back, and it was all good. (1046, a woman and kinship caregiver to a 3-year-old boy).

Caregivers shared that the learning environment was central to the CPP-FC, with one caregiver stating, “At the very beginning, it was really the connecting with the other foster parents really seeing their experiences, seeing them talk, and how they’ve handled scenarios that really helped to relate to the experiences that we were going through” (1056, a man and licensed caregiver to a 5-year-old boy). The value of learning from and sharing with other caregivers reflected the concept of caregivers as experts that is core to the CPP, saying “It’s good to see how other people do things where we can utilize those ideas and also being able to share different ideas with people” (1057, a man and licensed caregiver to an 8-year-old boy). Caregivers also unanimously shared positive feelings about group leaders: “I think they [group leaders] were awesome. They were very engaging… their involvement was good… like how they would also use their own personal stories with their own children” (1057, a man and licensed caregiver to an 8-year-old boy).

Finally, caregivers recognized the value of online delivery to support participation. One interviewee shared, “I usually do the trainings through [another organization] which is in-person. So, this is really my first time doing something like this… I really enjoyed it” (1022, a woman and licensed caregiver to a 5-year-old girl).

These ideas were consistent with what caregivers shared in surveys, where group leaders were consistently positively rated: 86% of responses rated group leaders as “Very Helpful,” and 14% rated them as “Helpful” or “A little Helpful.” Similarly, group discussions were rated consistently positive, with 78% of responses across all sessions indicating group discussions were “Very Helpful” and 22% indicating they were “Helpful” or “A Little Helpful.”

#### Theme 2: Caregiver and Child Factors Impact Experience with the CPP-FC

All participants reported being comfortable initiating the CPP-FC when children first entered their homes. Consistent with the logic model for the CPP (Breitenstein et al., 2020; Gross, n.d.), caregivers were motivated to participate because they wanted to address child behaviors. One caregiver stated, “We start[ed] seeing behaviors right off the bat because we already had some concerns and questions about the things that were going on… We did ask the physician, and she kind of pointed us to your direction (1014, a woman and licensed caregiver to a 4-year-old girl).” Consistent with the perspectives of the advisory board, caregivers also acknowledged that the range of behaviors observed and addressed by the CPP-FC was reflective of their experiences. One caregiver noted this as a strength that should be better communicated in advance, stating, “It’s not just one specific thing that you’re working on… put it in the materials [sign up information], and that way they [caregivers] know it’s multiple different things could help out with behavior.” (1014, a woman and licensed caregiver to a 4-year-old girl).

Caregivers also identified that changes made to tailor the CPP to foster and kinship care helped motivate them to participate. These included fulfillment of training hours as well as wanting to learn more as new caregivers of a child in their homes.

They told me that the hours that I spent doing this would count to renew my license. So that definitely does help how you know it’s working towards something in counting towards that. (1054, a woman and licensed caregiver to a 2-year-old girl)

When I went to the foster care clinic, a lady asked me if I wanted to participate. Once she told me that I get training hours for it, I was all in because I was almost to my deadline. (1022, a woman and licensed caregiver to a 5-year-old girl)

The desire to benefit and learn from peers, which is a core component of the CPP, was highlighted by some caregivers who wanted more interaction. “[A benefit of CPP-FC was] more hands-on stuff… like the role plays instead of just sitting there having the watch a bunch of different videos. […] When [discussing] the reward system, have [caregivers] write out a reward system, get class feedback, and then come back the next week and share how it worked.” (1014, a woman and licensed caregiver to a 4-year-old girl).

In contrast, other caregivers did not appreciate the direct engagement during weekly sessions and did not want to do activities outside of them.

For other classes, they don’t call on us… I feel like you should let a person volunteer sometimes. I mean I got used to it. But yeah, I wasn’t at first because I’m not used to being called on like that. (1049, a woman and licensed caregiver to a 7-year-old girl)

I, in particular, don’t like the extra work [referring to practice assignments], I’d rather do everything right in class, and because it’s just time-consuming, it’s not hard work but like I said when you gotta do homework and bath and all this stuff, you gotta do sometimes it’s just not time, and then you want your weekend to be free. (1051, a woman and licensed caregiver to a 5-year-old girl)

The pressure to participate and complete practice assignments may be complicated by the timing of recruitment into the CPP-FC, where caregivers are adjusting to new children in their homes. Despite detailed communication at the time of recruitment and in emails and text messages before initiating sessions, one caregiver reported, “I didn’t know how many classes it would be. I didn’t realize it was going to be once a week.” (1049, a woman and licensed caregiver to a 7-year-old girl). This may have contributed to decreased engagement with the program over time. One caregiver noted, “By the third class, it was only two of us… I had to participate a whole lot.” (1022, a woman and licensed caregiver to a 5-year-old girl).

Foster and kinship caregivers reported in surveys that the program was applicable to them (*M* across all sessions = 9.29, *SD* = 1.13, Observed range = 4–10) and that they would “Highly Recommend” (56%) or “Recommend” (44%) the program to others, further reflecting the idea that the CPP-FC addressed their unique experiences.

#### Theme 3: One Less Thing to Worry About—Making Attendance Possible

The modifications to the CPP program model for delivery, which included an online format and normalizing support for caregivers to simultaneously engage with the CPP-FC content and meet the needs of the children in their homes were valued by caregivers, who recognized that program changes enhanced their capacity to participate. “I thought they made it easy. I thought they made it simple for us, you know, just by being on Zoom, just by having virtual calls like this. With the amount of stuff we deal with or the amount of kids- we have 4 kids in sports, and we’re just all over the place Monday through Friday. So, I thought it was great being able to log in and communicate while we’re sitting there at practice. So, for me, I thought it was great.” (1057, a man and licensed caregiver to an 8-year-old boy).

Caregivers generally liked participating online. As one caregiver noted, “Doing it online was more convenient than having to go somewhere and participate” (1054, a woman and licensed caregiver to a 2-year-old girl). However, being home was not without challenges, particularly when one caregiver managed both class participation and the children in the home.

The only thing that kind of like kinda got me was because I was either handling something that was more important with the childrens that I had to deal with. (1014, a woman and licensed caregiver to a 4-year-old girl)

If [my husband] was in the office that day, he wouldn’t get home till 6 o’clock. I had three kids yelling in the background while I’m trying to focus. […] It was a little distracting if he was not here. (1054, a woman and licensed caregiver to a 2-year-old girl)

Sometimes, other family members provided childcare necessary to attend classes, with one caregiver saying, “My son has [been]… helping me out too with the kids” (1051, a woman and licensed caregiver to a 5-year-old girl). CPP-FC facilitated the offloading of some household burdens, following further adaptations to the program model for delivery. This included sending meals and care packages containing age-appropriate activities for children. Caregivers viewed those resources as helpful in supporting their active participation in the session.

The kids with the with the little activities and toys- the kids really loved opening those, and they were different. You had a variety of different things for different ages. (1054, a woman and licensed caregiver to a 2-year-old girl)

The pizza was definitely helpful. It allowed me not to have to prepare a meal, so that helped. (1022, a woman and licensed caregiver to a 5-year-old girl)

I’m going to class ‘cause I don’t have to cook dinner tonight. (1051, a woman and licensed caregiver to a 5-year-old girl)

At the end of the program, 67% found attending the group to be “Very helpful” and 33% found it “A little helpful.” Caregivers were “Very Satisfied” (67%) or “Satisfied” (33%) with CPP-FC.

#### Theme 4: Caregivers Found the Materials Applicable

Caregivers described the course content as helpful, applicable, and beneficial because it allowed them to learn new skills they had not previously been exposed to. One caregiver shared that the CPP-FC was “very much helpful […] I do that little exercise (referring to a stress management strategy taught in the program) and then come back to the situation and handle it different than what I was doing.” (1014, a woman and licensed caregiver to a 4-year-old girl).

New skills taught in the CPP-FC also supported caregivers with implementing routines and setting expectations with the children in their homes. Caregivers reported seeing successes early into the program because of that session being taught first. “It was very beneficial. It was something that I knew that I had to work on. Oh, the routine, just the nightly routine, helps the morning move more smoothly, and that’s something that I worked on throughout the program. It’s one of our very first sessions and I did see improvement in our morning routine.” (1022, a woman and licensed caregiver to a 5-year-old girl).

Caregivers also reported seeing positive behavior changes after implementing the concepts taught in session 1 of the CPP-FC.

The older one has learned our routines and our rules. He has stopped doing the things that he knows that we ignore or that we don’t like, or that he gets a timeout for. (1054, a woman and licensed caregiver to a 2-year-old girl)

She is more organized and willing to help around the house. She kind of knows what’s expected of her and does it. (1022, a woman and licensed caregiver to a 5-year-old girl)

Caregivers also valued the changes to the CPP-FC that contextualized children’s behaviors and helped them understand the connection between some of those behaviors and the backgrounds children in foster and kinship care have. One caregiver noted, “It helped me understand why she acted the way she did.” (1022, a woman and licensed caregiver to a 5-year-old girl). Another caregiver appreciated that the CPP-FC laid a foundation for communicating expectations that reminded them to be consistent. “And understanding children, that it [a boundary or limit] is gonna have to be reiterated on a regular basis.” (1057, a man and licensed caregiver to an 8-year-old boy). This understanding seemed to help some caregivers recognize where they can re-frame their approach to caring for the children in their homes.

I did end up learning a lot of things that I didn’t know or that I can improve on, things to make my life easier as a parent. And you know, just understanding behaviors and why kids act the way they do. (1022, a woman and licensed caregiver to a 5-year-old girl)

I think just realizing that foster kids come from a different background, and there’s trauma in their life and just thinking about that before disciplining or teaching. You know, just taking that into account has been helpful and just thinking about what would work best for this situation. (1054, a woman and licensed caregiver to a 2-year-old girl)

However, benefits were not universally observed. Caregivers of children with special needs noted that they needed more support than was offered by this prevention program.

I had reservations because I have an autistic child and I thought it was geared toward just a whole broad foster care... It really wasn’t so much to help him, but it did help with his younger siblings. (1051, a woman and licensed caregiver to a 5-year-old girl)

Not with [child]… she had to take medication and was in [a behavior treatment] program. She had been through a lot. (1049, a woman and licensed caregiver to a 7-year-old girl)

Importantly, some original materials from the CPP were recognized as helpful by caregivers, particularly in managing caregiver stress. This provided additional confirmation that some components of the CPP designed for parents also resonated with caregivers.

I was like, okay, I can put the kids to bed early, and I can spend time just by myself watching the TV show that I like instead of Coco Melon. So, it actually was very helpful for me. (1054, a woman and licensed caregiver to a 2-year-old girl)

The different ways of dealing with stress and how to calm down, to utilize some of those things, cause when you got 10 kids in the house, you can get to that level fast. So just being able to know some of the relaxing or to get your blood pressure back down and stress relief tactics that they taught us. And even the one where the one can leave the room, you know when so the other one can respond. That was helpful too. I think I’ve learned some good things. (1057, a man and licensed caregiver to an 8-year-old boy)

It taught us to have an adult time out, get away from the situation and think of how bad was the problem, was it something that you really could adjust? Let it pass. And not get so upset. (1051, a woman and licensed caregiver to a 5-year-old girl)

Consistent with themes from the qualitative interviews, caregivers rated program components positively. Topics discussed in sessions were mostly perceived to be “Very helpful” (81%), with 19% indicating discussions were “Helpful” or “A little helpful.” Videos were also rated as “Very helpful” (64%) by most caregivers, with 36% rating them as “Helpful” or “A little helpful” in weekly ratings and “Very useful” (75%) or “A little useful” (25%) in end-of-program surveys. Practice assignments were rated as “Very helpful” (75%) or “Helpful” (25%) in weekly ratings and as “Very helpful” (67%), “A little helpful” (22%), or “Not at all helpful” (11%) at the end of CPP-FC.

## Discussion

This study applied an existing conceptual approach to adapting evidence-based programs to new cultural contexts (Card et al., 2011) to a prevention program initially designed to support parents (Breitenstein et al., 2020) and tailored to support foster and kinship caregivers. The seven steps outlined by Card et al. were systematically followed in collaboration with an advisory board of foster and kinship caregivers and then evaluated using a mixed methods approach with foster and kinship caregivers who participated in the adapted intervention (CPP-FC). Advisory board members collaborated with investigators to identify core components of the program model and materials that required tailoring. Areas of tailoring included concepts that substantially differed for foster and kinship caregivers, such as the need to establish new routines with the children in their homes, incorporate families of origin, and acknowledge the influence of the past on child behaviors. These program model adaptations move beyond simply updating terminology; they reflect the unique aspects of caregiving that are unlikely to be included in programs designed for parents and children in parental care. Caregivers generally approved of and felt positive about the CPP-FC, referencing multiple aspects of the adapted program as helpful or beneficial when they discussed their experiences with it. This provided evidence to support the success of tailoring the CPP for the foster and kinship caregiving contexts as outlined by Card et al. (2011) and supporting the study’s expectations that applying the Card et al. framework would lead to high acceptability of CPP-FC.

### Implications for the CPP-FC

There were several areas where caregivers identified challenges with engagement, including more clear communication about group expectations at the outset of the program, needing additional resources and activities to address particularly challenging behaviors among children, and recognizing the reality that caregivers are participating in the program while also meeting other demands. These findings were brought back to the advisory board, where (1) recruitment materials were modified to better describe the CPP-FC; (2) group leader materials were further revised to include more details regarding trauma and behavior challenges for children in foster care that they can use to enhance discussion during weekly sessions; and (3) ground rules were added to normalize caregiver divided attention and disruptions while also supporting engagement with materials. This includes providing portable tablets to caregivers who can use their headsets to listen and attend to groups while moving around their home environment or in other settings (e.g., while in the lobby of visitation centers, in parking lots during sports practices), recording weekly sessions so caregivers can go back to content they may have missed while they stepped away from the group, and offering tips to help caregivers anticipate challenges they might encounter with remote participation (e.g., children’s behaviors) and identify solutions to try ahead of time.

### Implications for Prevention Programs in Foster and Kinship Care

Importantly, this study provides a framework that others could follow when adapting similar preventive interventions to families with foster and kinship care involvement. Given the lack of evidence-based programs adapted for child welfare contexts (see Supplemental Table 1), where only 40% of programs eligible for Title IV-E funding are rated as highly relevant to the child welfare system, more programs need to be adapted for those Title IV-E funds to achieve maximal impact. The framework outlined by Card et al. (2011) provides an achievable roadmap that can lead to effective adaptation of services specifically to the child welfare context, creating additional opportunities to enhance access to evidence-based preventive services and ensuring that those programs are maximally effective.

There are several limitations to the current study. First, the number of participants was small (*N* = 12), and the research design was not intended to produce generalizable findings. More research will be needed that draws on larger sample sizes and methods designed to determine generalizability to address important questions about whether prevention programs tailored to specific populations such as foster and kinship caregivers contribute to enhanced outcomes (e.g., observed reductions in caregiver stress, increased caregiver confidence and self-efficacy, reductions in child behavior problems). To address this limitation, the CPP-FC will be evaluated in a randomized clinical trial (Beal et al., 2024).

## Conclusions

This study provides preliminary evidence of the effectiveness of applying a rigorous approach to tailoring parenting prevention programs on the acceptability of those programs for special populations; in this case, drawing on the framework of Card et al. (Card et al., 2011) to adapt the CPP (Breitenstein et al., 2020) for foster and kinship caregivers. Results suggest that following the seven critical steps outlined by Card et al. resulted in a tailored intervention, CPP-FC, that was acceptable to and highly favored by caregivers, who reported high rates of satisfaction with program components and described how adapted aspects of the program were useful to them as they supported the behavioral wellbeing of young people in foster and kinship care. Similar approaches could be applied to other prevention programs to enhance acceptability for foster and kinship care, maximizing impact for these vulnerable children.

## Supplementary Information

Below is the link to the electronic supplementary material.

Supplementary file1 (DOCX 55 KB)

## Declarations

### Clinical Trial Registration

This study is registered under NCT06170047.
