# Supplementary material for: Tailoring the Chicago Parent Program for Foster and Kinship Caregivers: a Mixed Methods Approach
Source: Prev Sci. 2025 Mar 1;26(3):377–90. doi: 10.1007/s11121-025-01797-9 (PMC12064461; doi:10.1007/s11121-025-01797-9)
Supplement: Supplementary file 1 — Supplementary file1 (DOCX 55 KB) [file 11121_2025_1797_MOESM1_ESM.docx]

**Supplemental Table 1.**

Interventions Identified by the California Evidence-Based Clearinghouse (The California Evidence-Based Clearinghouse, 2024).

| **Search Criteria** | | | | |
| --- | --- | --- | --- | --- |
| **Scientific Rating** | | Well-supported by (1), Supported by (2), or Promising (3) Research Evidence | | |
| **Child Welfare System Relevance Level** | | High (1), Medium (2), and Low (3) | | |
| **Child Welfare Outcomes** | | Child/Family Well-Being | | |
| **Topic Area** | | Parent Training Programs that Address Behavior Problems in Children and Adolescents | | |
| **Age of Child** | | 2-8 years old | | |
| **Program Delivery Options** | | Adoptive Home, Birth Family Home, Foster/Kinship Care | | |
| **Results** | | | | |
| **Program Name** | **Scientific Rating** | | **Child Welfare Relevance** | **Program Delivery Options** |
| Promoting First Relationships | 2: Supported by research evidence | | 1: High | Adoptive, Birth Family, Foster/Kinship Care |
| DCCTF’s Effective Black Parenting Program | 3: Promising research evidence | | 1: High | Birth Family, Foster/Kinship Care |
| Early Pathways Program | 3: Promising research evidence | | 1: High | Adoptive, Birth Family, Foster/Kinship Care |
| Trust-Based Relational Intervention | 3: Promising research evidence | | 1: High | Adoptive, Birth Family, Foster/Kinship Care |
| Family Check-Up | 1: Well-supported by research evidence | | 2: Medium | Adoptive, Birth Family, Foster/Kinship Care |
|  |  | |  |  |
| Triple P | 1: Well-supported by research evidence | | 2: Medium | Adoptive, Birth Family, Foster/Kinship Care |
| 1-2-3 Magic | 3: Promising research evidence | | 2: Medium | Adoptive, Birth Family, Foster/Kinship Care |
| Incredible Years | 3: Promising research evidence | | 2: Medium | Adoptive, Birth Family, Foster/Kinship Care |
| Parent-Child Care | 3: Promising research evidence | | 2: Medium | Adoptive, Birth Family, Foster/Kinship Care |
| Systematic Training for Effective Parenting | 3: Promising research evidence | | 2: Medium | Adoptive, Birth Family, Foster/Kinship Care |
| Generation PMTO | 1: Well-supported by research evidence | | 1: High | Adoptive, Birth Family |

**Supplemental Table 2.** Qualitative Interview Guide

| **Introduction/warm-up:** |
| --- |
| 1. Tell me a little bit about your experiences in foster and kinship care and your relationship with the children in your home. 2. Is there anything from the Caregivers on Point program that stood out to you? What was the biggest surprise about the program? |
| **Reasons for Participating and Experiences with the Recruitment Process** |
| 1. When we talked to you in the clinic about Caregivers on Point, what was the process of learning about the program like? What concerns, questions, or reservations did you have before agreeing to participate? 2. What made you want to participate in this program when you initially learned about it? (only if the caregiver can’t think of anything - ex: money, training hours, child behavior strategies?) 3. What would you change about the enrollment (sign-up) process that would make it better? |
| **Convenience of Time and Site** |
| 1. What did you think of the day and time the group was scheduled? 2. How easy or hard was it for you to join the sessions? 3. Were there any barriers to participating in sessions or completing surveys? (childcare, technology, appointments). If the caregiver does not bring up the child behavior, ask about the child behavior specifically. 4. Are there any ways our team could have helped support you in CPP activities? 5. How helpful was us sending food and the comfort and play kits while you were participating in sessions? What would have been more helpful? (if can’t think of anything--tablets for children to interact with/watch while the caregiver was on the call, meals, different toys, separate programming for kids, etc.) 6. In terms of communication and follow-up that our team did with you, how did it feel when we reached out to you to complete surveys, attend sessions, etc. |
| **Group Dynamic in Sessions** |
| 1. Tell me 2 things about the sessions that you liked the most? 2. Tell me 2 things about the sessions that you liked the least? 3. Name one or two things that you noticed about the group leaders? 4. How were your interactions with the other caregivers in the program? 5. How did you feel about coming to sessions each week? (excited, not excited, indifferent?) What made you *(feeling caregiver gave*) about attending sessions? |
| **Program Content/Relevance** |
| 1. What was it like for you to start this program towards the beginning of placement for the child in your home? 2. Now, I’m going to recap each session. For each session, please tell me one or two things that stood out to you? 3. *For caregivers who had children transition out of their care during sessions*: How relevant did sessions afterward feel? Follow-up prompt: were you able to fully participate in the practices and group discussions? 4. Name 1-2 ways that the program impacted the way that you parent the children in your care? 5. What have you shared with others that you learned during the program. Tell me more—who did you share with? What did you share? Why did you share it? |
| **Caregiver and Child Changes** |
| 1. What changes within yourself have you noticed since you participated in the group? (Stress levels, managing child behaviors?) 2. What changes in the children in your home’s behavior have you seen since your participation in the program? 3. What social connections/relationships have you created or seen improvements in since participation in the program? |
| **Kinship Caregivers (if applicable to respondent)** |
| 1. How was your experience of being a kinship caregiver in the context of this program? 2. How could we have supported you better in this environment? |
| **Overall Feedback** |
| 1. What other suggestions do you have to improve this program or how it is delivered? If you were running the program, what would you do differently? |

**Supplemental Table 3.** Combined STROBE, SPIRIT, and SRQR checklist for this non-randomized, mixed-methods, single arm open intervention study.

|  | **SRQR** | | **STROBE** | **Recommendation** | | | **Page** |
| --- | --- | --- | --- | --- | --- | --- | --- |
|  | **Item No** | | **Item No** |  |  |  |  |
| **Title and abstract** | S1 | | 1 | (*a*) Indicate the study’s design, methods, and topic with a commonly used term in the title or the abstract | | | 1, 7 |
|  | S2 | |  | (*b*) Provide in the abstract an informative and balanced summary of what was done and what was found; include background, purpose, methods, results, and conclusions | | | 2 |
| **Introduction** | | | | | | | |
| Background/rationale | S3 | | 2 | Explain the scientific background and rationale for the investigation being reported/significance of the problem studied | | | 3 |
| Objectives | S4 | | 3 | State specific purpose/objectives, including any prespecified hypotheses if appropriate | | | 4, 5 |
| **Methods** | | | | | | | |
| Study design | -- | | 4 | Present key elements of study design early in the paper | | | 5 |
| Setting/Context | S7 | | 5 | Describe the setting, locations, and relevant dates, including periods of recruitment, exposure, follow-up, and data collection | | | 6 |
| Human Subjects Protection | S9 | | -- | Document approval by ethics review board and participant consent, confidentiality | | | 16, 17 |
| Researcher characteristics | S6 | | -- | Researchers’ characteristics that may influence the research; interactions between researchers’ characteristics and the research questions, approach, methods, results, and/or transferability | | | N/A |
| Participants | S8 | | 6 | (*a*) Give the eligibility criteria, sampling strategy, and the sources and methods of selection of participants. Describe methods of follow-up | | | 6 |
|  | -- | |  | (*b*) For matched studies, give matching criteria and number of exposed and unexposed | | | N/A |
| Variables | S11 | | 7 | Clearly define all outcomes, exposures, predictors, potential confounders, and effect modifiers. Give diagnostic criteria, if applicable. Include how data was collected and a description of instruments | | | 6,7 |
| Data sources/ measurement | S10 | | 8* | For each variable of interest, give sources of data and details of methods of assessment (measurement). Describe comparability of assessment methods if there is more than one group. Describe types of data collected and rationale. | | | 6,7 |
| Bias | S15 | | 9 | Describe any efforts to address potential sources of bias, enhance trustworthiness and credibility | | | N/A |
| Study size | -- | | 10 | Explain how the study size was arrived at | | | 6 |
| Quantitative variables | -- | | 11 | Explain how quantitative variables were handled in the analyses. If applicable, describe which groupings were chosen and why | | | 6,7 |
| Qualitative approach and research paradigm | S5 | | -- | Qualitative approach and guided theory if appropriate, identifying research paradigm and rationale | | | 7 |
| Data Processing | S13 | | -- | Methods for processing data prior to and during analysis, including transcription, data entry, data management, and de-identification | | | 6,7 |
| Statistical methods | S14 | | 12 | (*a*) Describe all methods, including those used to control for confounding | | | 7 |
|  |  |  |  | (*b*) Describe any methods used to examine subgroups and interactions | | | 6 |
|  |  |  |  | (*c*) Explain how missing data were addressed | | | N/A |
|  |  |  |  | (*d*) If applicable, explain how loss to follow-up was addressed | | | N/A |
|  |  |  |  | (*e*) Describe any sensitivity analyses | | | N/A |
| **Results** | | | | | | | |
| Participants | S12 | | 13* | (a) Report numbers of individuals at each stage of study—eg numbers potentially eligible, examined for eligibility, confirmed eligible, included in the study, completing follow-up, and analysed | | | 7 |
|  |  |  |  | (b) Give reasons for non-participation at each stage | | | N/A |
|  |  |  |  | (c) Consider use of a flow diagram | | | N/A |
| Descriptive data |  |  | 14* | (a) Give characteristics of study participants (eg demographic, clinical, social) and information on exposures and potential confounders | | | 8 |
|  |  |  |  | (b) Indicate number of participants with missing data for each variable of interest | | | 8 |
|  |  |  |  | (c) Summarise follow-up time (eg, average and total amount) | | | N/A |
| Outcome data | -- | | 15* | Report numbers of outcome events or summary measures over time, Main findings with links to empirical evidence | | | 7, 8 |
| Main results | -- | | 16 | (*a*) Give unadjusted estimates and, if applicable, confounder-adjusted estimates and their precision (eg, 95% confidence interval). Make clear which confounders were adjusted for and why they were included | | | 8,9,10, 11 |
|  |  |  |  | (*b*) Report category boundaries when continuous variables were categorized | | | 8,9,10, 11 |
|  |  |  |  | (*c*) If relevant, consider translating estimates of relative risk into absolute risk for a meaningful time period | | | N/A |
|  | S16 & S17 | |  | Report main findings and evidence to substantiate | | | 8,9,10, 11 |
| Other analyses | -- | | 17 | Report other analyses done—eg analyses of subgroups and interactions, and sensitivity analyses | | | N/A |
| **Discussion** | | | | | | | |
| Key results | S18 | | 18 | Summarise key results with reference to study objectives | | | 13 |
| Limitations | S19, S20 | | 19 | Discuss limitations of the study, taking into account sources of potential bias or imprecision. Discuss both direction and magnitude of any potential bias. Include conflicts nf interest that might influence conclusions and how those were managed | | | 14 |
| Interpretation | S18 | | 20 | Give a cautious overall interpretation of results considering objectives, limitations, multiplicity of analyses, results from similar studies, and other relevant evidence | | | 14 |
| Generalisability | -- | | 21 | Discuss the generalisability (external validity) of the study results | | | 14 |
| **Other information** | | | | | | | |
| Funding | S21 | | 22 | Give the source of funding and the role of the funders for the present study and, if applicable, for the original study on which the present article is based | | | 15 |
| **Study Period** | | **Enrollment** | | **Post-Allocation** | | **Close-out** | |
| Timepoint | | Baseline  (t_0_) | | Sessions 1-11  (t_1_-t_11_) | Follow-up (t_12_) | Graduation (t_13_) | |
| Eligibility screen | | X | |  |  |  | |
| Informed consent | | X | |  |  |  | |
| Baseline assessment | | X | |  |  |  | |
| Demographic  Characteristics | | X | |  |  |  | |
| Weekly surveys | |  | | X |  |  | |
| Session Satisfaction | |  | | X |  |  | |
| Applicability to caregivers | |  | | X |  |  | |
| End-of-session surveys | |  | |  | X |  | |
| Program satisfaction | |  | |  | X |  | |
| Satisfaction with training  components | |  | |  | X |  | |
| Recommendations | |  | |  | X |  | |
| Qualitative interview | |  | |  | X |  | |
| Program graduation session | |  | |  |  | X | |

*Give information separately for exposed and unexposed groups.

**Note:** An Explanation and Elaboration article discusses each checklist item and gives methodological background and published examples of transparent reporting. The STROBE checklist is best used in conjunction with this article (freely available on the Web sites of PLoS Medicine at http://www.plosmedicine.org/, Annals of Internal Medicine at http://www.annals.org/, and Epidemiology at http://www.epidem.com/). Information on the STROBE Initiative is available at http://www.strobe-statement.org.
